# A resected case of acinar cell carcinoma of the pancreas with liver metastasis following chemotherapy using modified FOLFIRINOX

**DOI:** 10.1186/s40792-023-01729-1

**Published:** 2023-08-23

**Authors:** Shuhei Yamada, Haruka Motegi, Yoshiki Kurihara, Tomonori Shimbo, Isao Kikuchi, Toshiki Wakabayashi, Tsutomu Sato

**Affiliations:** Department of Gastroenterological Surgery, Akita City Hospital, 4-30 Kawamoto Matsuoka-machi, Akita-city, Akita, Japan

**Keywords:** Acinar cell carcinoma, Pancreas, Liver metastases, Chemotherapy, FOLFIRINOX

## Abstract

**Background:**

Acinar cell carcinoma of the pancreas is a rare exocrine malignancy representing less than 1% of all pancreatic neoplasms. It has been reported that it responds to treatment differently from pancreatic ductal adenocarcinoma and the treatment algorithm for acinar cell carcinoma usually depends on the stage of the respective tumor and the patient’s current status.

**Case presentation:**

A 60-year-old man presented with upper abdominal pain and anorexia. Abdominal ultrasonography showed a large-sized hepatic mass and he was referred to our hospital. Contrast-enhanced computed tomography demonstrated a 110-mm low-density area occupying the right hemi-liver and an enhanced mass of 70 × 56 mm in the tail of the pancreas, which seemed to directly infiltrate into the spleen. The case was diagnosed as acinar cell carcinoma with a simultaneous liver metastasis identified by liver biopsy. Upfront resection of pancreatic cancer with distant metastasis might not be considered as an optimal choice, and in this case chemotherapy was administered prior to curative resection. Chemotherapy using the modified FOLFIRINOX regimen was undertaken, resulting in a partial remission; the liver tumor reduced in size from 110 to 47 mm and the pancreatic tumor from 70 to 40 mm. The patient then safely underwent curative hepatic resection with distal pancreato-splenectomy. Histological examinations revealed small-sized atypical cells with large nuclei that had formed acinar patterns, and immunostaining with trypsin was positive in tumor cells, which was in accordance with acinar cell carcinoma. More than 3 years later, the patient is doing well without any recurrence.

**Conclusion:**

Aggressive and curative surgery in combination with chemotherapy such as FOLFIRINOX could be a treatment option to achieve long-term survival in cases of acinar cell carcinoma with liver metastases.

## Background

Acinar cell carcinoma of the pancreas (pACC) is a rare exocrine malignancy representing less than 1% of all pancreatic neoplasms. It has been reported that pACC responds to treatment differently from pancreatic ductal adenocarcinoma (PDAC) [1–3]. The treatment algorithm for pACC usually depends on the stage of the respective tumor and the patient’s present status because of the lack of evidence due to the small patient numbers. Although metastatic pancreatic tumors are generally considered to have a dismal prognosis, there has been quite few reports on aggressive multimodal treatment strategy in pACC patients. We herein report a case of pACC with a simultaneous hepatic metastasis, for which the preoperative chemotherapy with the modified FOLFIRINOX regimen was very effective. After undergoing curative resections, the patient is doing well without any recurrence for more than 3 years. The efficacy of FOLFIRINOX and aggressive surgery in cases of pACC with synchronous hepatic metastases is discussed.

## Case presentation

**Fig. 1:**
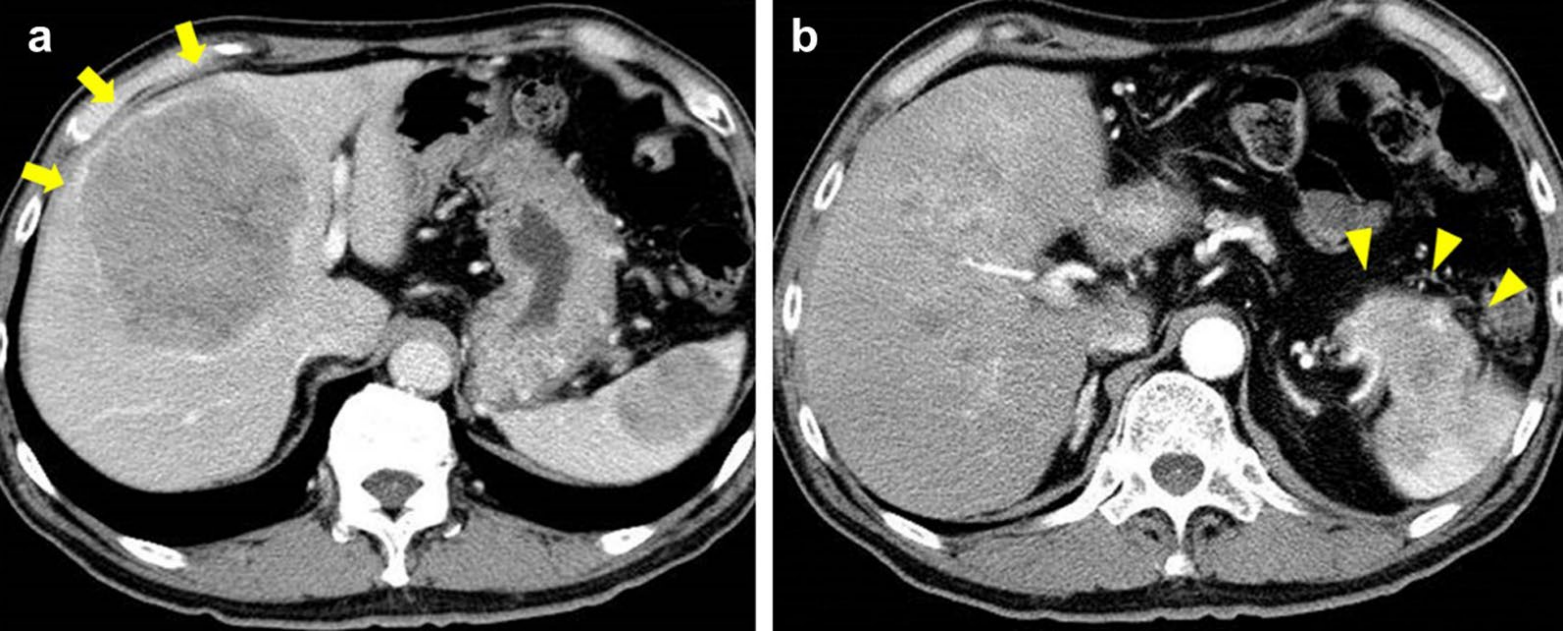
**a** An enhanced low-density area with a thin capsule was occupied the right hemi-liver. The size of the tumor was 110 × 90 mm (arrow); **b** an enhanced mass of 70 × 56 mm in the tail of the pancreas, which seemed directly infiltrated into the spleen (arrowhead)

A 60-year-old man was admitted to our hospital complaining of persistent upper abdominal pain associated with anorexia. The patient had a medical history of diabetes mellitus, hyperlipidemia and systemic hypertension. Abdominal ultrasonography (US) revealed large tumors in the liver and the pancreas. Contrast-enhanced computed tomography (CECT) demonstrated an enhanced low-density area with a thin capsule and a tumor 110 × 90 mm in size in the right hemi-liver (Fig. 1a). CECT also revealed an enhanced mass of 70 × 56 mm in the tail of the pancreas, which seemed to directly infiltrate into the spleen (Fig. 1b).

**Fig. 2:**
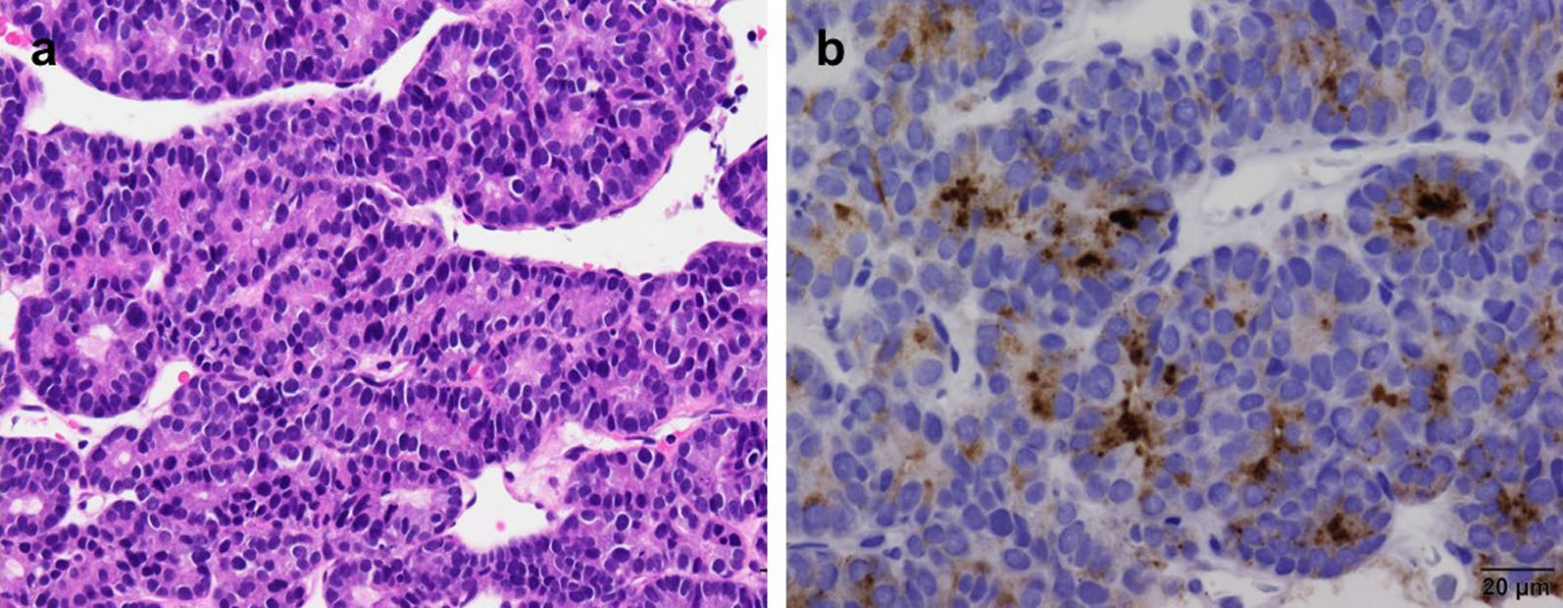
Histopathology of the liver tumor. **a** Small, atypical epithelia with increased nuclear chromatin had densely proliferated forming small glandular cavities (H&E 100 ×). **b** The cytoplasm was heterogeneously positive with trypsin, which supported the diagnosis of hepatic metastasis of pACC (trypsin 100 ×)

The disease was diagnosed as a pancreatic tumor with a hepatic metastasis, or vice versa, while the latter was considered more uncommon than the former. Both the liver and pancreas were available as target organs for tumor biopsy, and the liver was chosen in this case. Two liver specimens were obtained using an 18-gauge needle under US guidance. Histological examinations by hematoxylin and eosin (H&E) staining revealed that small-sized atypical cells with large nuclei had densely proliferated to form an acinar appearance (Fig. 2a). The tumor cells and their nuclei were consistent in size and acinar-like arrangement. Microscopic appearances were consistent with pACC, when immunostaining with trypsin was performed, and the cytoplasm was heterogeneously positive with trypsin as shown in Fig. 2b, which supported the diagnosis of hepatic metastasis of pACC. Tumor markers in the blood before chemotherapy were as follows; alpha-fetoprotein (AFP) 56.5 ng/ml [normal level: < 10 ng/ml], protein induced by vitamin K absence-II (PIVKA-II) 25 mAU/ml [normal level: < 40 mAU/ml], carcinoembryonic antigen (CEA) 2.3ng/ml [normal level: < 5.0 ng/ml], cancer antigen 19–9 (CA19-9) 34 U/ml [normal level: < 37 U/ml], duke pancreatic monoclonal antigen type 2 (DUPAN-2) 75 U/ml [normal level: < 150 U/mL], s-pancreas antigen-1 (SPAN-1) 27 U/ml [normal level: < 30 U/ml], neuron specific enolase (NSE) 23.6 ng/ml [normal level: < 16.3 ng/ml], and elastase 14,600 ng/dl [normal level: < 300 ng/dl]. AFP, NSE and elastase were higher than the normal range.

**Fig. 3:**
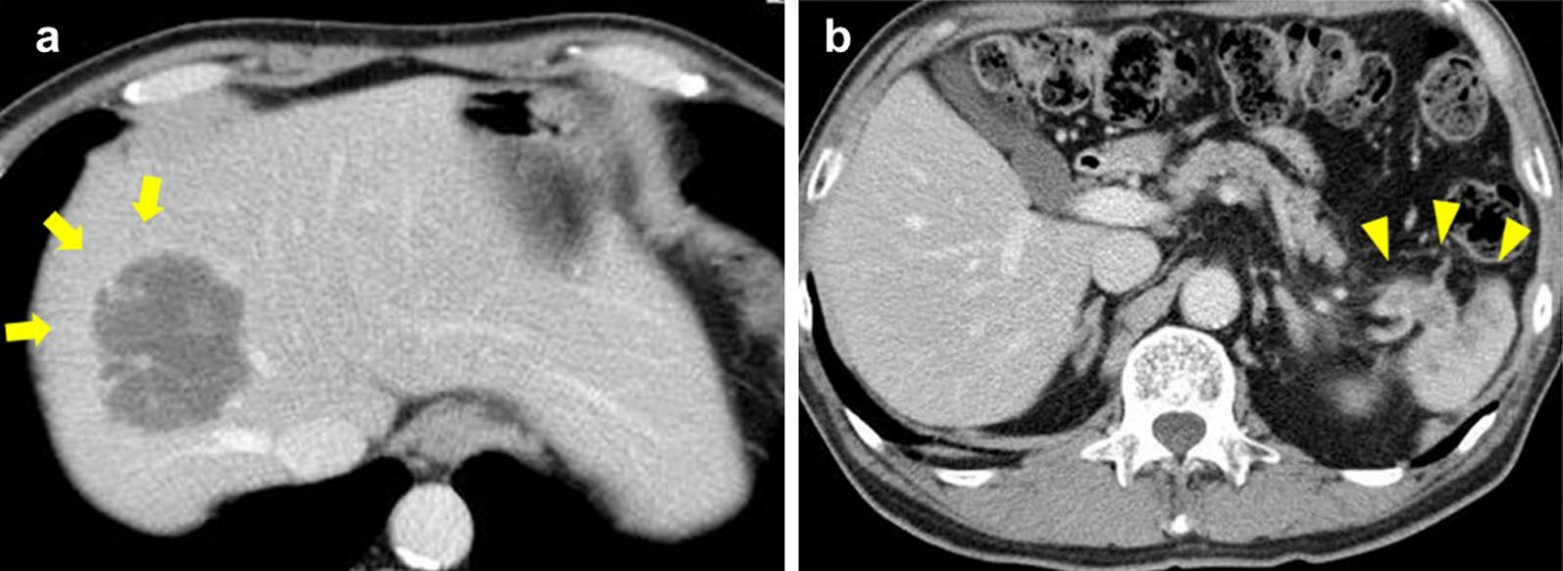
**a** After three courses of modified FOLFIRINOX, the tumors were markedly decreased in size. **a** The hepatic tumor had shrunk to 47 × 45 mm, which was nearly 50% of the original area (arrow). **b** The pancreatic tumor had shrunk to 40 × 36 mm, which was 57% of the original area (arrowhead)

Although both tumors in the liver and the pancreatic tail to the spleen could be removed concurrently, the surgical procedure was regarded as having certain comorbidity risks if they were removed simultaneously. Additionally, favorable results were not highly anticipated without a waiting period to observe the biological behavior of the tumor. For these reasons, chemotherapy was planned prior to the resections of the liver and the pancreas instead of upfront surgery, and the modified FOLFIRINOX regimen was selected. After three courses of modified FOLFIRINOX, the tumors were markedly decreased in size: the hepatic tumor had shrunk to 47 × 45 mm (43% of the original area); and the pancreatic tumor had shrunk to 40 × 36 mm (57%), as shown in Fig. 3a, b, respectively. It was determined through CECT that the patient had a partial response according to version 1.1 of the Response Evaluation Criteria in Solid Tumors. No newly developed tumors were identified, and the infiltration to the splenic hilum appeared negative after three courses of chemotherapy. Elevated tumor markers were all normalized before the surgery: AFP to 4.1 ng/ml, NSE to 1.7 ng/ml, and elastase to 175 ng/dl. The surgical procedure planned before chemotherapy was indicated in the patient.

Three weeks prior to resection, percutaneous transhepatic portal vein embolization of the right portal branch was performed to increase the safety of the surgical procedures. The estimated future remnant liver volume was increased from 45 to 55%. The patient underwent extended right hemi-hepatectomy including the middle hepatic vein followed by distal pancreato-splenectomy. To resect the left prerenal fascia and left adrenal gland with safety margins, we performed posterior radical antegrade modular pancreatosplenectomy with lymph node dissection. The operating time was 6 h and 15 min, and the operative blood loss was 417 ml. No blood transfusion was necessary during the procedure.

**Fig. 4:**
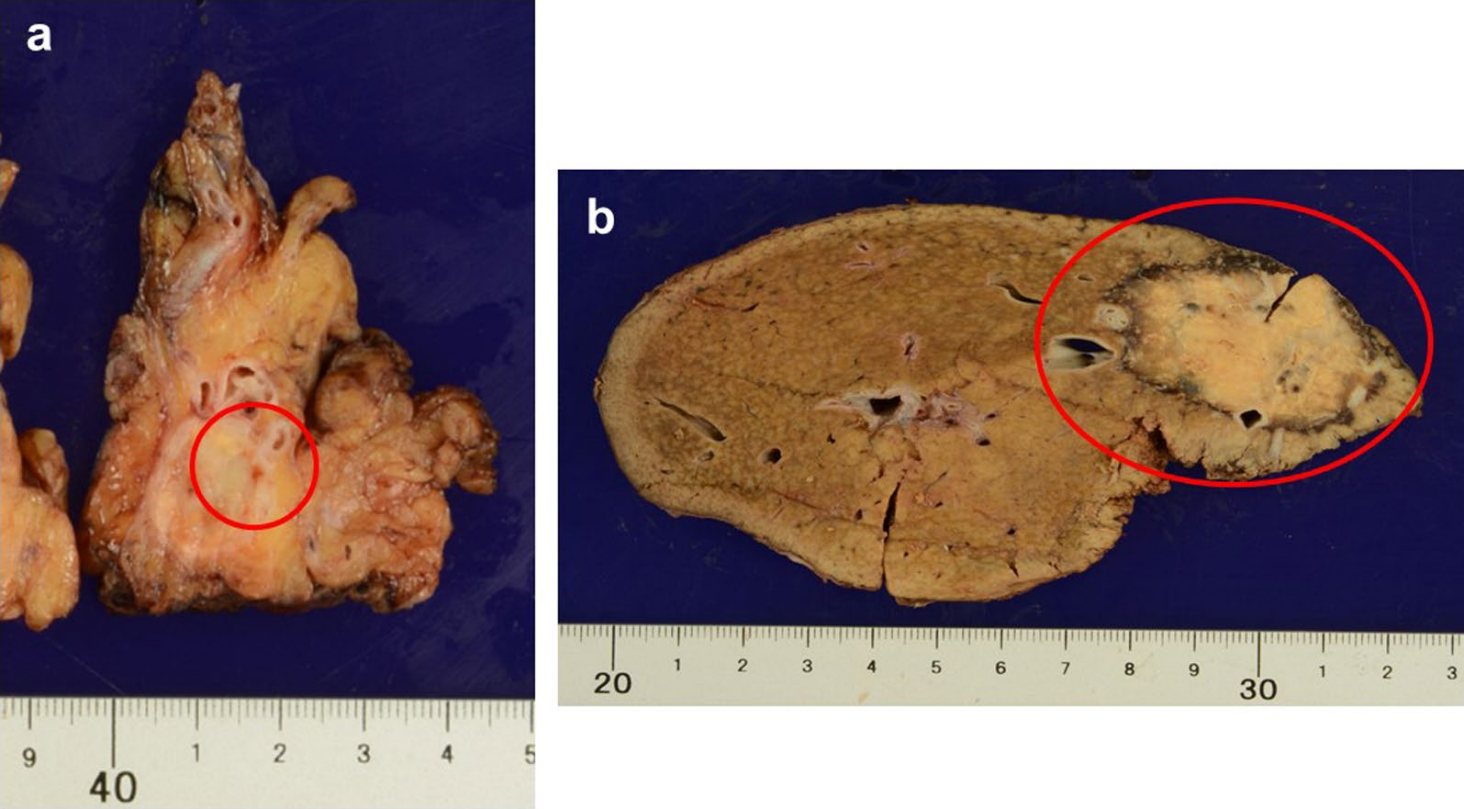
The extended right hemi-hepatectomy included the middle hepatic vein, and distal pancreatectomy and splenectomy with retroperitoneal tissue resection included left adrenal gland and Gerota’s fascia (tumor in the circle). **a** A 15-mm-sized tumor existed in the tail of pancreas, which was surrounded by consistently scarred tissues adjacent to the splenic hilum showing unclear boundaries. **b** A 45 × 25-mm-sized white-colored tumor with a thin capsule in the liver

As shown in Fig. 4a, b, the resected specimen of the liver revealed a 45 × 25 mm-sized white-colored tumor with a thin capsule in the liver. In the tail of the pancreas, a 15 mm-sized tumor was identified, which was surrounded by consistently scarred tissues adjacent to the splenic hilum that showed unclear boundaries.

**Fig. 5:**
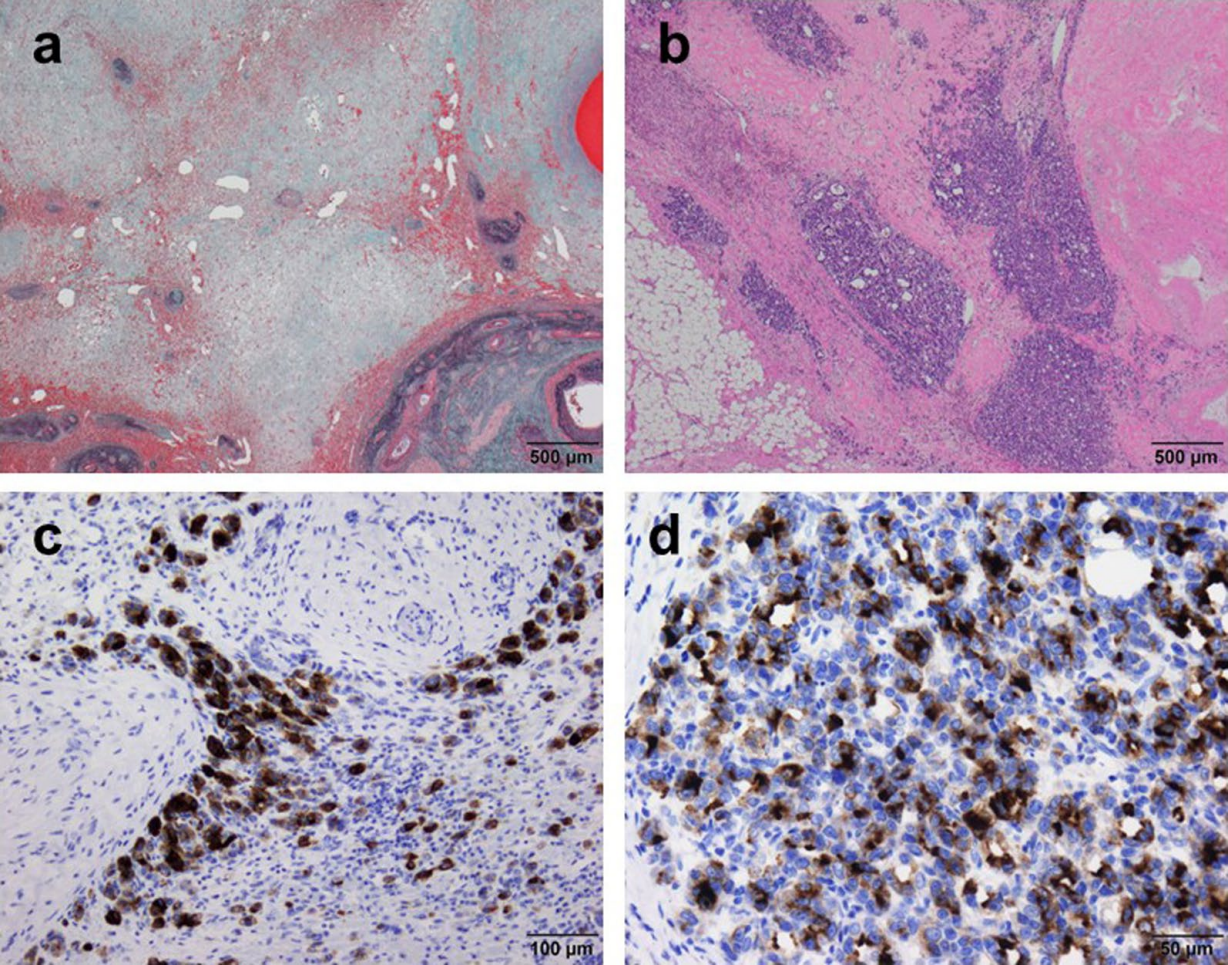
Histopathological features of the resected specimen. **a** Almost all the liver tumor was replaced by fibrous scarred tissues and tumor cells seemed no longer viable in the scarred area (Elastica Masson stain 20 ×). **b** The tumor cells in the pancreas were small-sized atypical cells with large nuclei forming acinar patterns. Acinar-like structures were more frequently observed in the pancreatic tumor than in the liver tumor (H&E 20 ×). **c** Tumor cells stained with trypsin in the liver tumor (trypsin 100 ×). **d** Tumor cells stained with trypsin in the pancreatic tumor (trypsin 100 ×)

Histological examination revealed that the majority of the liver tumor was replaced with fibrous scarred tissues, and tumor cells seemed no longer viable in the scarred area (Fig. 5a). The residual tumor cells seemed degenerative, and small-sized atypical cells with large nuclei formed cell clusters sporadically. Acinar-like arrangements and structures that were characteristic in the preoperative liver biopsy had disappeared. The histological appearance of the pancreatic tumor was composed of similar tumor cells to those of the liver tumor, while acinar-like structures were more frequently observed in the pancreatic tumor than in the liver tumor (Fig. 5b). According to the Evans grade, the efficacy of the chemotherapy was grade 3 for both hepatic and pancreatic specimens. As for the histological diagnosis, microscopic findings were consistent with those of pACC, with none having lymph node metastases. Immunostaining with trypsin revealed dense positivity in the cytoplasm (Fig. 5c, d). However, synaptophysin and chromogranin A were negative. These results supported the initial diagnosis of a hepatic metastasis of pACC. Not only shrinkage in size, but also histological degenerative changes demonstrated the excellent effects of FOLFIRINOX prior to surgery.

The postoperative course was uneventful, and the patient was discharged from the hospital on postoperative day 14. The patient was prescribed S-1 administration for 6 months as adjuvant chemotherapy. More than 3 years after the surgery, the patient is doing well without any recurrent tumors in the liver or in the local region.

## Discussion

pACC is a rare exocrine malignancy representing less than 1% of all pancreatic neoplasms[1] and accounted for 0.4% of all pancreatic tumors according to the Japanese Pancreatic Cancer Registry in 2007 [2]. The clinical features of pACC are different from PDAC. As far as tumor makers are concerned, levels of CEA, CA19-9, SPAN-1, and DUPAN-2 which are abnormally high in PDAC are low in pACC [2]. AFP is occasionally high [2, 4, 5] and NSE is elevated in cases of mixed acinar-cell endocrine carcinoma, in which endocrine cells immunohistochemically exceed 30% [6, 7]. Therefore, tumor makers are sometimes useful for differential diagnosis of pancreatic neoplasms. It has been frequently reported that pACC responds to treatment differently than PDAC. For instance, Schmidt et al. [3] compared 865 cases of pACC with 367,999 cases of PDAC, and demonstrated that the stage-specific 5-year survival rate in pACC was better than that in PDAC (pACC 40.2% vs. PDAC 9.8% at stage II). Generally, the prognosis of pACC is thought to be better than that of PDAC, however, some authors have reported that pACC has a high recurrence rate of more than 50% [8, 9].

Because of the shortage in case numbers, the treatment algorithm for pACC usually depends on the stage of the respective tumor and the patient’s present status. An aggressive approach with complete tumor resection is regarded as the best option when it is possible [10]. Previous reports have shown that aggressive resection with multimodal treatment for pACC leads to favorable prognosis (Table 1) [4, 11–19]. Among 12 cases, aggressive surgery was performed in 11 cases with liver metastases, and half of the cases were synchronous. Aggressive resection for liver metastases of pACC seem to be optimal treatment strategy. The previous report showed that pACC has genetic mutation in APC /ß-catenin which tend to be found in colorectal cancer, not in PDAC [20]. Thus, the characteristics of pACC might be similar to colorectal cancer for which resection of liver metastases improve survival with or without chemotherapy [21–24]. Patients with advanced disease may benefit from multimodal treatment including chemotherapy. As in other diseases, such as PDAC [25–27], primary systemic chemotherapy may be useful to reduce the tumor burden and allow the success of subsequent resections. Zong, et al. [28] reported that complete surgical resection for pACC even in advanced disease might improve survival. Due to the favorable prognosis, the 5-year survival of resected pACC with distant metastases was 50% and was not significantly different from stage II and III. However, unresected cases were associated with a poor prognosis. We should consider performing aggressive surgical curative resection with multimodal chemotherapy for pACC.

**Table 1:** Review of successful resection of pACC with distant metastases

Report	Synchronous or metachronous metastases	The site of metastases	Primary surgery for metastatic tumor	Preoperative chemotherapy	Clinical course	Reported survival
Butturini et al. [4]	Synchronous	Liver metastasis	Liver metastasis excision	Unspecified	Recurrence and death with primary disease	31 months (dead)
Butturini et al. [4]	Metachronous	Liver metastasis	Liver metastasis excision	gemcitabine	No recurrence	85 months
Armstrong et al. [11]	Synchronous	Solitary liver metastasis	RFA	No	Recurrence at liver treated with RFA and systemic chemotherapy (capecitabine, imatinib, etoposide, and doxorubicin), and at mesenteric lesions with systemic chemotherapy (sorafenib and temozolomide)	96 months (dead)
Cananzi et al. [12]	Synchronous	Five liver metastases	Right hemi-hepatectomy	Docetaxel, irinotecan and cetuximab	Recurrence at liver, para-aortic lymph node, peritoneal and retroperitoneal tissue, adrenal gland, and brain. Liver metastases were treated with surgery and RFA. The other metastases were all resected Newly detected liver metastases were treated with chemotherapy (nab-paclitaxel and panitumumab)	132 months
Di marco et al. [13]	Metachronous	Three liver metastases	RFA	Gemcitabine plus capecitabine	No recurrence	68 months
Di marco et al. [13]	Metachronous	Two liver metastases	RFA	Gemcitabine plus capecitabine	Recurrence at peritoneal tissue treated with systemic chemotherapy (capecitabine plus irinotecan, gemcitabine plus capecitabine)	86 months (dead)
Hashimoto et al. [14]	Synchronous	Two liver metastases	Left hemi-hepatectomy and partial resection	No	Recurrence at liver (partial liver resection and hepatic arterial infusion with 5-FU, cisplatin, and mitomycin C)	63 months
Jauch et al. [15]	Synchronous	Solitary liver metastasis	Right hemi-hepatectomy	Capecitabine plus oxaliplatin	Recurrence at liver (complete eradication by chemotherapy)	42 months
Maehira et al. [16]	Metachronous	Multiple liver metastases, local recurrence	The removal of multiple liver metastases and local recurrence in the pancreatic bed	Cisplatin plus irinotecan	No recurrence	57 months
Ohara et al. [17]	Metachronous	Solitary liver metastasis, rectum	Right posterior sectorectomy and abdominoperitoneal resection	No	No recurrence	40 months
Sumiyoshi et al. [18]	Synchronous	Peritoneal dissemination	Resection of disseminated nodule	No	No recurrence	73 months
Suzuki et al. [19]	Metachronous	Four liver metastases	Extended left hemi-hepatectomy and partial resection	No	Recurrence at liver (partial liver resection for solitary metastasis)	65 month

The efficacy of chemotherapy in treating pACC has not yet been established. Good responses have been observed in pACC patients treated with gemcitabine or 5-fluorouracil-based combination therapies with irinotecan, a platinum analog, docetaxel, or erlotinib [3, 29]. Most therapeutic regimens have been the same as those utilized for PDAC or colorectal carcinomas [30–34]. Yoo, et al. reported the efficacy of oxaliplatin-containing regimens in 15 patients with pACC [34], while Proquin et al. reported a case with prolonged survival with pACC treated by gemcitabine and oxaliplatin [33]. Although gemcitabine-based chemotherapy regimens are thought to be effective for pancreatic tumors, Takahashi et al. suggested that platinum- and/or irinotecan-containing regimens exhibited stronger efficacy in patients with pACC, and the overall survival tended to be better in patients who had received these types of chemotherapy compared with those who had not [29]. On the basis of these reports [29–32, 34], the modified FOLFIRINOX regimen was chosen as the first-line therapy in the present case. The efficacy and/or duration of preoperative chemotherapy for pACC is unknown. As mentioned above, complete resection for pACC might prolong survival, and we considered the present case to be technically resectable but oncologically unfavorable because of the high tumor burden. We selected three courses of neoadjuvant chemotherapy following the PDAC regimen previously reported [26, 35, 36]. Three courses of FOLFIRINOX were sufficient and tolerable for pancreatic and major hepatic resection. Prolonged periods of chemotherapy including irinotecan and/or oxaliplatin cause histopathological liver injury and increase postoperative morbidity and mortality [37–43]. The tumor response was excellent after three courses of FOLFIRINOX, as demonstrated in CECT scans as well as in histological examinations. The preceding chemotherapy apparently reduced the size of the tumors and made the surgical procedures, which consisted of right extended hemi-hepatectomy and distal pancreato-splenectomy, much safer and easier to perform. Additionally, the distance from the resection margin was sufficient, especially from the retroperitoneal tissues. Accordingly, the effectiveness of the chemotherapy may contribute to the long-term outcome of the patient, who was doing well without any recurrent tumors when this report was submitted. FOLFIRINOX has proven to be a highly effective chemotherapy regimen in the treatment of pACC with liver metastases. In accordance with the treatment protocol for PDAC, adjuvant chemotherapy with S-1 was administered in the present case [44] because the FOLFIRINOX regimen is highly effective in tumors such as PDAC. Although the efficacy of adjuvant chemotherapy was not established, some reports have described the efficacy of S-1 against pACC [18, 45, 46]. On the other hands, previous study showed that adjuvant chemotherapy seems to be effective only in a subgroup of poor prognostic patients in PDAC [47]. Considering the lower malignant potential of pACC, it might be necessary to select the patients who should undergo adjuvant treatment based on tumor stage and/or pathological type of pancreatic tumor. Effectiveness of adjuvant chemotherapy for pACC is very important topic. Several previous studies have reported the molecular abnormalities of pACC [30, 48, 49]. Of note, one case with the *BRAF V600E* driver mutation treated with targeted therapy using dabrafenib and trametinib achieved an almost complete remission of the tumors with prolonged clinical benefit [30]. Regarding the molecular characterization of pACC, various chromosomal imbalances have been identified including *EGFR, MGMT, HSP90,* and *L1CAM* alternations [50]. If possible, we should evaluate various genetic alternations and treat such patients with targeted therapy.

Above all, complete resection with chemotherapy for potentially resectable pACC might prolong survival and serve as the best treatment. We should consider curative resection with an aggressive approach.

## Conclusions

We experienced a rare case of simultaneous liver metastasis of pACC that could be treated by surgical excision. The modified FOLFIRINOX regimen was markedly effective as the preoperative treatment in this case. Our findings imply that aggressive and curative surgery in combination with an effective preceding chemotherapy such as modified FOLFIRINOX could be a treatment option to achieve long-term survival in cases of pACC with liver metastases.

## Data Availability

Not applicable.

## Abbreviations

pACC: Acinar cell carcinoma of the pancreas
PDAC: Pancreatic ductal adenocarcinoma
US: Ultrasonography
CECT: Contrast-enhanced computed tomography
H&E: Hematoxylin and eosin
AFP: Alpha-fetoprotein
PIVKA-II: Protein induced by vitamin K absence-II
CEA: Carcinoembryonic antigen
CA19-9: Cancer antigen 19-9
DUPAN-2: Duke pancreatic monoclonal antigen type 2
SPAN-1: S-pancreas antigen-1
NSE: Neuron specific enolase
